# Trends in the national early warning score are associated with subsequent mortality – A prospective three-centre observational study with 11,331 general ward patients

**DOI:** 10.1016/j.resplu.2022.100251

**Published:** 2022-05-21

**Authors:** Eetu Loisa, Antti Kallonen, Sanna Hoppu, Joonas Tirkkonen

**Affiliations:** a Medical School, Faculty of Medicine and Life Sciences, Tampere University, FI-33014 Tampere, Finland; b Department of Emergency, Anaesthesia and Pain Medicine and Emergency Medical Service, Tampere University Hospital, PO Box 2000, FI-33521 Tampere, Finland; c Faculty of Medicine and Health Technology, University of Tampere, FI-33014 Tampere, Finland; d Department of Emergency, Anaesthesia and Pain Medicine and Emergency Medical Service, Tampere University Hospital, PO Box 2000, FI-33521 Tampere, Finland; e Department of Intensive Care Medicine, Tampere University Hospital, PO Box 2000, FI-33521 Tampere, Finland

**Keywords:** National early warning score, Trend, Vital signs, Prevention of in-hospital cardiac arrest

## Abstract

**Aim:**

To investigate whether trends in the NEWS values are associated with patient mortality in general ward patients.

**Methods:**

A one-year prospective observational study in three hospitals in Finland. All data on patients’ NEWS values during the first three days of general ward admissions were collected. The linear regression model was used to investigate the association of the NEWS trajectories with subsequent mortality. We used three outcome measures: 4–7-day, 4–14-day and 4–21-day mortality rates after the 0–3 days of initial hospitalization, respectively.

**Results:**

The study cohort consisted of 11,331 general ward patients. The non-survivors had higher initial NEWS score values in all outcome categories (all p < 0.001). The non-survivors had a rising trajectory in their NEWS values in all the outcome categories, whereas the survivors had a downward trajectory in their NEWS values in all outcome categories (data presented as first- and third-day’s median values): an increase from 5.0 to 6.0 vs. a decrease from 1.5 to 1.0 (4–7-day non-survivors vs. survivors), an increase from 4.0 to 5.0 vs. a decrease from 1.5 to 1.0 (4–14-day non-survivors vs. survivors) and an increase from 4.0 to 5.0 vs. a decrease from 1.5 to 1.0 (4–21-day non-survivors vs. survivors). In the linear regression model, these differences in trends were statistically significant in all the outcome categories (p < 0.05).

**Conclusion:**

The NEWS score trajectory during the first three days of general ward admission is associated with patient outcome. Further studies are warranted to determine specific thresholds for clinically relevant changes in the NEWS trajectories.

## Introduction

Following trends in hospitalized patients’ vital signs is of utmost importance in order to recognize the individuals that are not responding to treatments from the vast majority of patients that are getting better.1., 2., 3. Today, early warning scores, such as the national early warning score (NEWS), have been widely internationally adopted to everyday practice across prehospital emergency services, emergency departments and general wards to enable early detection of critically ill patients.4., 5. However, near all studies investigating early warning scores, or vital signs in general, have only used a single set of values (measured at a predefined time point, such as the emergency room arrival) when investigating their association with subsequent mortality and morbidity.6., 7. Despite routine vital signs measurements provide general ward staff with trajectories on their patients’ improvement/deterioration, published data on how these changes on patients’ vital signs over time are associated with outcomes in everyday practice, are very scarce. Indeed, a recent systematic review concluded that there is an apparent lack of high-quality evidence regarding trends in vital signs and subsequent morbidity.^8^

The statistical association of single NEWS values and subsequent adverse patient outcomes is well established.9., 10. We, however, hypothesized that the trajectories in hospitalized general ward patients’ NEWS values during the first three days of admission represent patients’ improvement or silent deterioration. Thus, the aim of this study was to investigate whether trends in the NEWS values are associated with patient mortality in a large, prospective three-centre trial.

## Methods

### Study design and setting

We conducted this one-year, prospective observational cohort study in three hospitals in Tampere, Finland. These three hospitals (Tampere University Hospital (Tays), and two regional hospitals Valkeakoski Hospital (VALS) and Hatanpää Hospital (HASA) provide secondary- and tertiary level in-hospital care for the Pirkanmaa Hospital district and include all medical and surgical specialties. The Pirkanmaa hospital district provides hospital services for a catchment population of 530,000 citizens and the most advanced care (neurosurgery etc.) for of 900,000 citizens.

All study hospitals have implemented the NEWS system for the follow-up of patients’ vital signs.^11^ Supplement A presents the NEWS system in detail. The NEWS2 system was published in 2017;^12^ it has an alternative blood oxygen saturation scale for those patients with confirmed hypercapnic respiratory failure. Because the NEWS2 has failed to show any benefits as compared with the original NEWS and raised concerns of its feasibility altogether,13., 14., 15. the study sites use the original NEWS. As the available resources differ between the hospitals on on-call time, the responses for patient deterioration are hospital-specific. While Tays and HASA have medical emergency teams, VALS has a response team operating from the emergency department.

The study hospitals begun implementing the Medanets® mobile solution system in emergency departments and general wards in 2016. The mobile solution app enables bedside recording of all clinical measurements. The system automatically calculates patient’s current NEWS, shows preceding NEWSs and trends, and records all the measurements and NEWS to the hospital’s electronic vital signs datasheet. The app demands that all vital signs are measured before it provides further data for the user. The frequency of the measurements is individualized according to patient’s condition; the minimum frequency of vital signs measurement is every 12 h. The app does not alert hospital’s emergency teams or treating physicians, rather the purpose of the system is to facilitate bedside nursing work and standardize patient follow-up.

### Participants

The 45 general wards (with altogether approximately 800 beds) in the three study hospitals that implemented the Medanets® mobile solution system at least four months before the study period (1.1.2019–31.12.2019) begun, were involved. Emergency departments, intensive care wards, high dependency units, operation rooms & post-anaesthetic units, and general wards that had not implemented/ were still in the implementation phase with the mobile solution system, were excluded. This study only included adult patients (≥18 years old) that were admitted for at least three consecutive days to the above defined wards.

### Data collection

As stated above, the NEWS measurements were conducted as part of nurses’ normal clinical routines. For the study purposes, the recordings were also prospectively collected to a separate database in the Pirkanmaa Hospital District’s internal secure data server. All the datasets were time- and ward-labeled and included patients’ social security numbers that include information on age and gender. Mortality data up to 90 days were derived from Digital and Population Data Services Agency in Finland, as mortality data for those patients that die after their hospital discharge may take 30–60 days to be updated to the system.

### Outcomes

In-hospital mortality among normal general ward patients is low, and adverse events occurring within 24 hours of random patient evaluation are extremely rare.16., 17., 18. In order to capture clinically relevant deaths with an incidence enabling reliable statistical analyses, we used three distinct outcome measures (4–7-day, 4–14-day and 4–21-day mortality after the 0–3 days of initial hospitalization) so that both short- and longer-term mortalities were captured.

### Statistical analysis

Statistical programming was done with Python version 3.8.6 and R version 3.6.3. Data are represented as counts and percentages and continuous data as medians and percentiles. To date, there is no universal consensus on how NEWS trends should be studied and interpreted,^8^ and many previously used methods to investigate trends include several weaknesses.^2^ In general ward setting it seems to require ≥ 12-hour follow-up in order to set apart natural variation within stable vital signs from real trend with prognostic value.^19^ We hypothesized a reliable trend among normal general ward patients could be derived from a three-day follow-up of vital signs. The median NEWS values per day were determined and represented the score for the day concerned (first, second and third day, respectively).

The Kruskal-Wallis Test was used to investigate the difference in the very first NEWS values between the survivors and non-survivors. Linear regression analyses were performed in order to examine the NEWS trends in the first three days and subsequent mortality. Slopes of the regression models were evaluated with null hypothesis of zero slope using Wald Test and t-distribution of the test statistic.^20^ Additional two-dimensional distribution estimate for visualization purposes of the score distribution on the temporal-score axis was done using Gaussian kernel density estimate. Kernel density estimation is a way to estimate the probability density function of a random variable in a non-parametric way.^21^ All tests were two-sided and p-value of < 0.05 was considered statistically significant.

### Ethical considerations

All data were collected to a Pirkanmaa Hospital District’s secure database and handled anonymously as per the European Union general data protection regulation 2016/679 (GDPR). The Ethics Committee of the Pirkanmaa Hospital District approved the study protocol (Approval number R20007R) and waived the need for informed consent. The general characteristics of this trial were recorded into an international database as part of a larger study set (ClinicalTrials.gov Identifier: NCT04055350). The STROBE checklist for observational studies was followed.^22^

## Results

### Study cohort

**Fig. 1:**
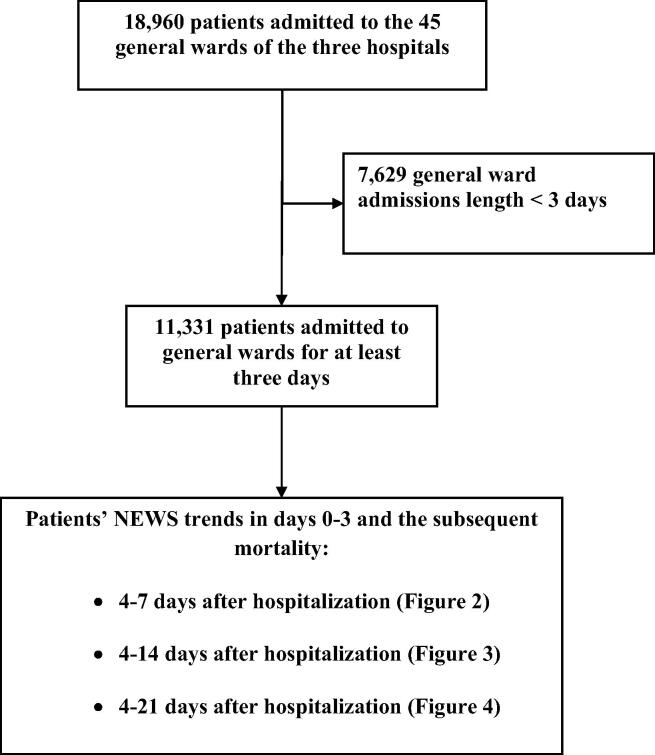
The final study cohort. NEWS, National Early Warning Score.

The initial cohort consisted of almost 19,000 patients admitting to general wards of the tree hospitals. After exclusion of general ward admissions with duration less than three days (n = 7,629), the final cohort consisted of 11,331 patients. Fig. 1 presents the formation of the final study cohort.

### Patient demographics

Median [Q1, Q3] age of the study cohort was 72 [61,81]. Majority (53%) of the patients were male. Most of the patients (81%) admitted to university hospital and approximately one third (32%) had surgical reason for admission. Table 1 presents patient demographics for the whole study cohort.

**Table 1:** Patient demographics and outcomes.

Patient characteristics			
	Age (years)	72	[61, 81]
	Sex (male)	5,974	(53)
	University hospital	9,128	(81)
	Admission ward (Medical)	7,783	(69)
First measured NEWS on general ward*		1.5	[0, 3]
Outcome			
	Died 4-7 days after hospitalization	110	(1.0)
	Died 4-14 days after hospitalization	250	(2.2)
	Died 4-21 days after hospitalization	344	(3.0)

Continuous variables are given as median [Q_1_, Q_3_]. Other data are presented as counts (percentages). *NEWS at the beginning of the general ward admission. NEWS, National Early Warning Score.

### Patient outcomes and initial NEWS values

Table 1 presents patient outcomes for the whole study cohort. The mortality rates were 1.0% (n = 110) for 4–7 days, 2.2% (n = 250) for 4–14 days and 3.0% (n = 344) for 4–21 days after the hospitalization and the three initial ward days.

As expected, the non-survivors had higher initial NEWS values upon general ward arrival (the very first measured values on general wards) than did the survivors (median 5 [2.50, 6.88] vs. 1 [0, 3], p < 0.001 for mortality on days 4–7; 42., 6. vs. 1 [0, 3], p < 0.001 for mortality on days 4–14; and 3.52., 5. vs. 1 [0, 3] p < 0.001 for mortality on days 4–21).

### Association of NEWS trend with subsequent mortality

The non-survivors had an increasing trend (from 5.0 to 6.0 for 4–7-day mortality, from 4.0 to 5.0 for 4–14-day mortality; and from 4.0 to 5.0 for 4–21-day mortality) between their median first- and third-day’s NEWS values, whereas the survivors had a decreasing trend (from 1.5 to 1.0 in all outcome categories) between their median first- and third-day’s NEWS values.

**Fig. 2:**
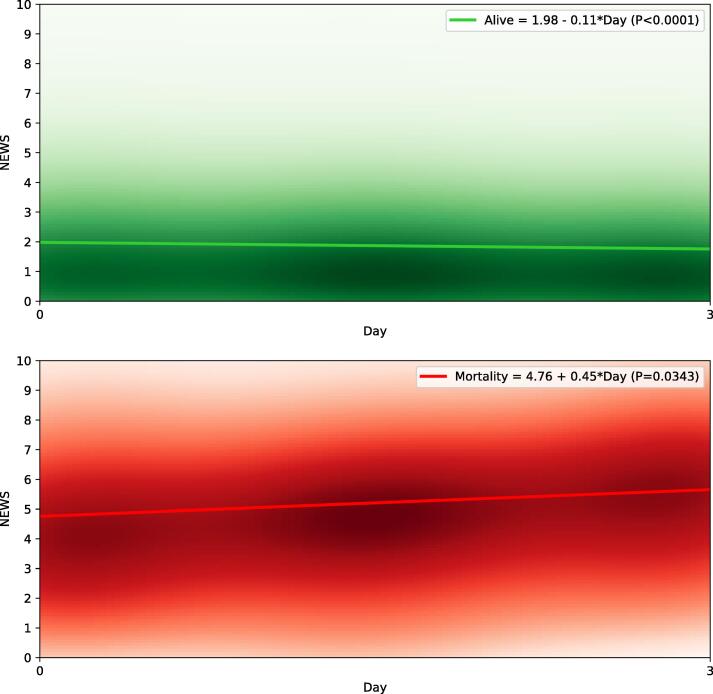
NEWS score evolution of the first three days after admission and association with 4–7 days mortality. Linear regression models with Kernel density estimates are demonstrated separately for 7-day survivors (green) and non-survivor (red). The intensifying color presents the increasing rate of patients scoring a certain NEWS value. NEWS, National Early Warning Score.

**Fig. 3:**
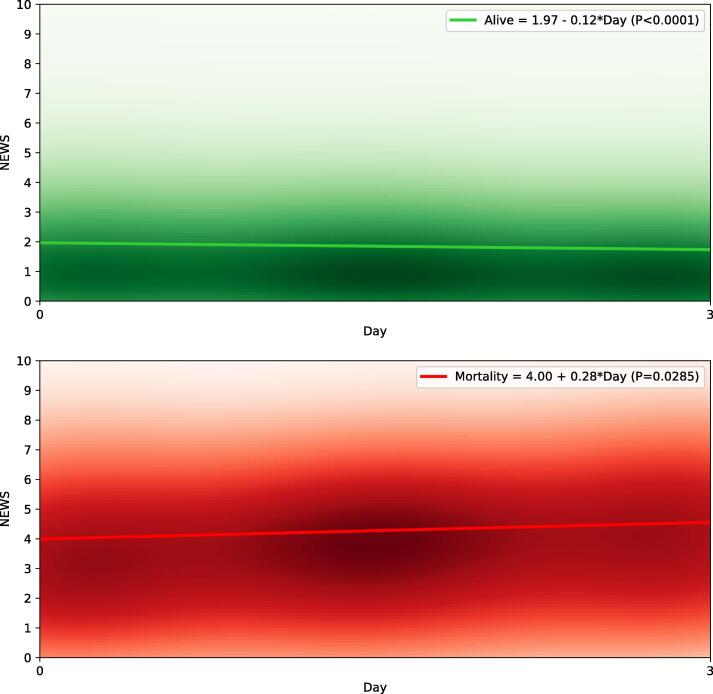
NEWS score evolution of the first three days after admission and association with 4–14 days mortality. Linear regression models with Kernel density estimates are demonstrated separately for 14-day survivors (green) and non-survivor (red). The intensifying color presents the increasing rate of patients scoring a certain NEWS value. NEWS, National Early Warning Score.

**Fig. 4:**
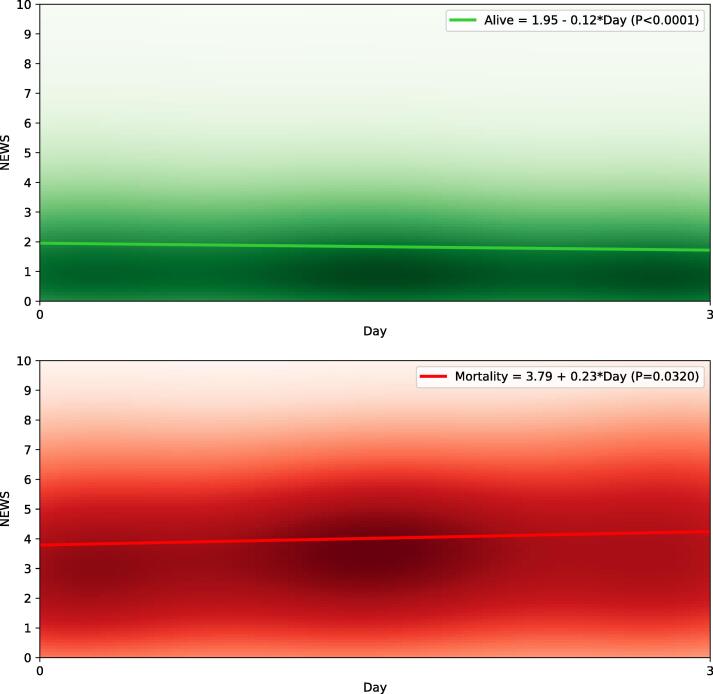
NEWS score evolution of the first three days after admission and association with 4–21 days mortality. Linear regression models with Kernel density estimates are demonstrated separately for 21-day survivors (green) and non-survivor (red). The intensifying color presents the increasing rate of patients scoring a certain NEWS value. NEWS, National Early Warning Score.

The NEWS score evolution for the survivors and non-survivors are demonstrated separately for 4–7-day mortality, 4–14-day mortality and 4–21-mortality with the linear regression models and Kernel density estimates in Fig. 2, Fig. 3, Fig. 4, respectively. The Kernel density estimates show that there is overlap in the groups (i.e., some survivors have increasing NEWS trend whereas some non-survivors have decreasing NEWS trend), as could be expected. In the linear regression model, the non-survivors had a rising trajectory in their NEWS values in all the outcome categories (4.76 + 0.45*Day, p = 0.034 for 4–7 day mortality; 4.00 + 0.28*Day, p = 0.029 for 4–14 day mortality; 3.79 + 0.23*Day p = 0.032 for 4–21 day mortality), whereas the survivors had a downward trajectory in their NEWS values in all the outcome categories (1.98–0.11*Day p < 0.001 for 4–7 day mortality; 1.97–0.12*Day p < 0.001 for 4–14 day mortality; 1.95–0.12*Day p < 0.001 for 4–21 day mortality).

## Discussion

### Key findings

In this prospective, three-centre, one-year cohort study we verified for the first time that increasing NEWS score values during the first three days after admission are associated with mortality, whereas survivors on average have a decreasing trend in their NEWS-values.

### Prognostic value of NEWS score evolution

It is clinically plausible that trends in NEWS values present patient deterioration or improvement. A few previous studies on trends in single vital parameters over variable time frames have documented the association of trends and mortality in non-critical care settings.2., 19., 23., 24. Observation of trends over time is indeed essential, since many of the patients eventually facing severe adverse event do not have abnormal vital signs or high NEWS score on admission.1., 19. Thus, observation of trend may assist in identification of these patients.^19^ Indeed, focusing on the trends instead of single values may increase the accuracy of prognostication^2^ and even marginal increase in the score (or failure to improve initially high score) may be associated with subsequent mortality.25., 26., 27.

Even though deteriorating patients tend to have increasing NEWS scores, it is obvious that not every patient follows this averaged rule. Some of the patients suffering adverse outcomes have unchanged / even decreasing NEWS prior the event.19., 28., 29. As demonstrated well before, and in this trial also, the initial high scores undoubtable predict mortality.6., 9., 10. Patients initially scoring high NEWS should probably show a fast trend of stabilization. Vice versa, patients initially scoring very low NEWS likely are at lower risk even though the trend is slightly increasing.^24^

### From universal thresholds to individualized assessment utilizing trend?

Since the implementation of NEWS and other early warning scores (EWSs), studies have attempted to define certain one-size-fits-all-thresholds in EWS values that should lead to automated escalation of care or RRT activation, but this has not resulted in breakthrough due to several problems.^30^ First, there is some published data presenting that similar (N)EWS deviations are not equally associated with mortality in different patient populations. For instance, only minor deviations are associated with mortality in the elderly as compared with younger patients,31., 32., 33., 34., 35. patients with chronic hypoxemia fulfil the desaturation thresholds all the time,36., 37. and EWS does not necessarily perform equally well between surgical and medical patients.38., 39. Second, overly sensitive (low) EWS thresholds for single measurements may lead rapid response team over-utilization.38., 40. These facts make optimal thresholds for single EWS measurements not universal but rather institution- and resource-dependent.

The NEWS is a tool for the nursing staff to recognize patient deterioration in time. However, in several cases there is probably a need for individualized assessment of NEWS values by treating physicians, taking into account the patient specific variables.^41^ The NEWS trend should be one of the aspects considered. Indeed, although Chester et al. found that individual measurements are not very sensitive to predict deterioration in elderly patients, deviation from the one’s own baseline is more accurate to identify deterioration.^42^ In addition to utilizing the trend of the NEWS, future research could explore the information content of the two-dimensional nonparametric distribution in the temporal domain to identify thresholds from the Gaussian manifold that could exhibit prognostic value in accurately detecting deteriorating patients as early as possible.

### Strengths and limitations of the study

Despite lack of evidence, the trends in patients’ NEWS are constantly followed as a part of routine clinical practice. To our knowledge, this is the first study to confirm the association of trends in NEWS score evolution with patient mortality – and in a large, prospective, multicentre setting. Due to the system used in the study hospitals, we captured full vital signs datasets and encountered no missing data. The cohort consisted of over 11,000 patients of highly heterogeneous general ward population and the data capture happened in a pragmatic way.

This study was of observational design; associations with mortality in cohort studies do not *per se* translate into causality. A general limitation inherent in all studies investigating vital signs’ trends over time is that measurements are not made in fixed time points in real life. Further, there is no standardized method defining ‘a trend’.^8^ Our study is no exception to these limitations. It must also be clearly stated that our study investigated associations of averaged trends in a large patient cohort; with this current data we are unable to set clear clinical decision rules to be utilized bedside and thus our results do not provide guidance for individualized patient care. Future studies should focus on defining clinically applicable thresholds for NEWS trends.

Further limitations include the facts 1) that we are unable to comment whether some trends or single high NEWSs were acted upon, or deliberately not acted upon if a patient had ‘do not resuscitate / not for intensive care’ order 2) this study was conducted in Finnish health care system and the results may not apply globally.^43^

## Conclusions

The NEWS score trajectory during the first three days of general ward admission is associated with mortality and survival. However, future studies are warranted to clearly define clinically relevant thresholds for increasing and decreasing trends in the NEWS.

Conflict of interest

None.

### CRediT authorship contribution statement

**Eetu Loisa:** Conceptualization, Methodology, Writing – original draft, Writing – review & editing. **Antti Kallonen:** Methodology, Formal analysis, Data curation, Writing – review & editing, Visualization. **Sanna Hoppu:** Writing – review & editing, Supervision, Project administration. **Joonas Tirkkonen:** Conceptualization, Methodology, Writing – original draft, Writing – review & editing, Supervision, Project administration.
