# Supplementary material for: Trends in the national early warning score are associated with subsequent mortality – A prospective three-centre observational study with 11,331 general ward patients
Source: Resusc Plus. 2022 May 21;10:100251. doi: 10.1016/j.resplu.2022.100251 (PMC9127395; doi:10.1016/j.resplu.2022.100251)
Supplement: Supplementary data 1 [file mmc1.pdf]

Supplement A. National Early Warning Score (NEWS) according to The Royal College of Physicians.

	3	2	1	0	1	2	3
Respiratory rate (breaths/min)	≤8		9—11	12—20		21—24	≥25
Blood oxygen saturation (%)	≤91	92—93	94—95	≥96			
Any supplementary oxygen		Yes		No			
Temperature (°C)	<35.0		35.1—36.0	36.1—38.0	38.1—39.0	≥39.1	
Systolic blood pressure (mmHg)	≤90	91—100	101—110	111—219			≥220
Heart rate (beats/min)	≤40		41—50	51—90	91—110	111—130	≥131
A, V, P, U				A			V, P, U

Alert (A), Voice (V), Pain (P), Unresponsive (U)
